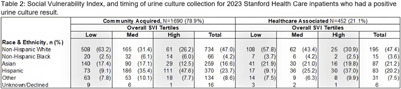# Exploring Social Vulnerability and Urine Culture Studies in Hospitalized Adult Patients in a Single Center, Northern California, 2023

**DOI:** 10.1017/ash.2025.341

**Published:** 2025-09-24

**Authors:** Mindy Sampson, Nidia Rodriguez-Ormaza, Eugenia Miranti, Guillermo Rodriguez Nava, Jorge Salinas

**Affiliations:** 1 Stanford University; 2 Stanford Health Care; 3 Stanford University School of Medicine

## Abstract

**Background:** Racial and ethnic disparities have been demonstrated across a range of healthcare outcomes and services, including infectious disease burden. Individuals who reside in communities with increased social vulnerability are more likely to experience worse infection-related outcomes. These disparities are likely exacerbated by structural and systemic inequities experienced during inpatient care, even for common diagnoses, such as urinary tract infections. We explored the relationship between race, population-level social vulnerability, and urine culture results in the inpatient setting. **Methods:** We conducted a retrospective cohort study at Stanford Health Care from January–December, 2023. We included all adult inpatients who had a urine culture collected. The Center for Disease Control’s Social Vulnerability Index (SVI) was used as a composite measure of social vulnerability. Patient addresses from electronic health records (EHR) were geocoded to determine census tract-level SVI designation using the California-specific SVI database, and out-of-state addresses were excluded. Unhoused patients were identified by a discrete field in the EHR. We included demographics, urine culture results, and time of urine culture collection. Community-associated positive urine cultures were defined by a collection time ≤48 hours from admission, while healthcare-associated positive cultures were collected >48 hours from admission. **Results:** There were 5,374 admissions with urine cultures collected in 2023. The overall median SVI was 0.34. Compared to the statewide median overall vulnerability of 0.50[IQR: 0.25–0.75], our inpatient population resided in less vulnerable areas. When comparing patients with positive and negative urine cultures, the overall SVI, the four-specific SVI themes and SVI quartiles were similar. Unhoused patients were more likely to have a negative culture than a positive culture. Patients who identified as Asian were more likely to have a healthcare-associated positive urine culture than a community-associated positive culture. Patients who identified as Hispanic were more likely to have a community-associated positive culture than a healthcare-associated positive urine culture. Patients who identify as white or black had similar likelihood of developing a community-associated or healthcare-associated positive culture. **Discussion:** We did not find any differences in SVI among patients based on urine culture positivity. However, when stratified by community- vs healthcare-associated we found that patients who identify as Asian or Hispanic may be more likely to have a positive urine culture. These differences in outcomes are likely complex and multifaceted, potentially related to various social drivers of health present both before and during admission. Further exploration is needed to understand what is contributing to these findings.

**Figure:**
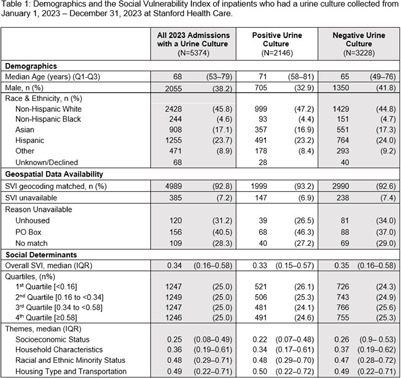

**Figure:**